# Potassium Starvation in Yeast: Mechanisms of Homeostasis Revealed by Mathematical Modeling

**DOI:** 10.1371/journal.pcbi.1002548

**Published:** 2012-06-21

**Authors:** Matthias Kahm, Clara Navarrete, Vicent Llopis-Torregrosa, Rito Herrera, Lina Barreto, Lynne Yenush, Joaquin Ariño, Jose Ramos, Maik Kschischo

**Affiliations:** 1 Department of Mathematics and Technology, RheinAhrCampus, University of Applied Sciences, Koblenz, Remagen, Germany; 2 Department of Microbiology, Campus de Rabanales, University of Córdoba, Córdoba, Spain; 3 Instituto de Biologia Molecular y Celular de Plantas UPV-CSIC, Ciudad Politécnica de la Innovación, Universidad Politécnica de Valencia, Valencia, Spain; 4 Institut de Biotecnologia I Biomedicina & Department of Biochemistry and Molecular Biology, Universitat Autònoma de Barcelona, Cerdanyola del Vallès, Barcelona, Spain; Stockholm University, Sweden

## Abstract

The intrinsic ability of cells to adapt to a wide range of environmental conditions is a fundamental process required for survival. Potassium is the most abundant cation in living cells and is required for essential cellular processes, including the regulation of cell volume, pH and protein synthesis. Yeast cells can grow from low micromolar to molar potassium concentrations and utilize sophisticated control mechanisms to keep the internal potassium concentration in a viable range. We developed a mathematical model for *Saccharomyces cerevisiae* to explore the complex interplay between biophysical forces and molecular regulation facilitating potassium homeostasis. By using a novel inference method (“the reverse tracking algorithm”) we predicted and then verified experimentally that the main regulators under conditions of potassium starvation are proton fluxes responding to changes of potassium concentrations. In contrast to the prevailing view, we show that regulation of the main potassium transport systems (Trk1,2 and Nha1) in the plasma membrane is not sufficient to achieve homeostasis.

## Author Summary

Without potassium, all living cells will die; it has to be present in sufficient amounts for the proper function of most cell types. Disturbances in potassium levels in animal cells result in potentially fatal conditions and it is also an essential nutrient for plants and fungi. Cells have developed effective mechanisms for surviving under adverse environmental conditions of low external potassium. The question is how. Using the eukaryotic model organism, baker's yeast (*Saccharomyces cerevisiae*), we modeled how potassium homeostasis takes place. This is because, through mathematical modeling and experimentation, we found that the electro-chemical forces regulating potassium concentrations are coupled to proton fluxes, which respond to external conditions in order to maintain a viable potassium level within the cells. Our results challenge the current understanding of potassium homeostasis in baker's yeast, and could potentially be extended to other microorganisms, including non-conventional yeasts such as the pathogenic *Candida albicans*, and plant cells. In the future, the fundamental bases for this descriptive and predictive model might contribute to the development of new treatments for fungal infections, or developments in crop sciences.

## Introduction

Potassium is an essential cation required for many cellular processes including the regulation of cell volume, intracellular pH, protein synthesis, activation of enzymes, and maintenance of the plasma membrane potential [1]–[4]. In their natural environment, most cell types have to accumulate intracellular potassium against a strong concentration gradient. Animal cells utilize the energy stored in ATP to directly pump potassium ions into the cell via the 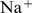/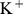 ATPase. This enzyme is absent in most fungi and plants [2], which have developed alternative mechanisms to control the intracellular potassium concentration. *Saccharomyces cerevisiae (S.c.)* cells can grow in media with a potassium concentration ranging from 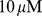 to 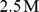. Despite extensive knowledge about the identity and function of most potassium transporters in this organism [3], a systems level understanding of the interplay and regulation of the various transport pathways is still lacking.

In *S.c.*, uptake of potassium across the plasma membrane is driven by the membrane potential, which itself is generated by proton pumping via the 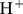-ATPase, Pma1 [5], [6]. The high affinity and high velocity transporter, Trk1, is the major uptake system for potassium. The expression levels of the other Trk protein, Trk2, are low, compared to Trk1, and therefore considered of minor importance [7], [8]. A low affinity uptake observed by electrophysiological techniques in *trk1,2* double mutants has been attributed to the putative calcium blocked channel Nsc1, though the gene responsible for this transport activity has not been found yet [9], [10]. Efflux of potassium is strongly pH-dependent and coupled to sodium toxicity. The antiporter Nha1 extrudes 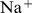 or 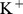 ions in exchange for protons under acidic environmental conditions and contributes to the continuous cyclic flux of potassium ions across the plasma membrane and to pH regulation [11], [12]. It is only at higher external pH that potassium or sodium is actively extruded by the Ena1 ATPase [13]–[15]. Another potassium efflux system is the voltage gated channel, Tok1. Electrophysiological studies revealed that Tok1 opens at positive membrane potentials, which do not occur under normal physiological conditions [16]. Potassium is also stored in intracellular compartments, in particular in the vacuole. The effect of intracellular transport is, however, not sufficiently characterized yet [3], [17].

Besides protons, a number of other ions are associated with the transport of potassium. The anion bicarbonate was shown to be important for potassium accumulation [18]. Decarboxylation reactions produce carbon dioxide, which is quickly converted to carbonic acid (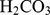), by carbonic anhydrase. Carbonic acid can either diffuse freely across the cell membrane or dissociate into bicarbonate (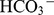), and protons. While protons can be extruded via Pma1, the permeability of bicarbonate is very low compared to that of carbonic acid. The resulting accumulation of bicarbonate provides the link to potassium homeostasis; the negative charges carried by bicarbonate can be balanced by potassium cations. In principle, other weak acids could contribute in a similar way to potassium accumulation, but our results below and previous investigations suggest that the bicarbonate reaction plays an important role [18]. Potassium transport is also related to ammonium toxicity [19]. Under low external potassium conditions, ammonium leaks into the cells, presumably via potassium transporters. Toxic concentrations of ammonium are counteracted by increased production and excretion of amino acids [19].

The maintenance of a minimal potassium concentration requires the orchestration of the different transport systems under the constraints of various thermodynamic forces. In this article, we use a mathematical model in conjunction with a novel inference algorithm (the reverse tracking algorithm) and model-driven experimentation to identify the key transport mechanisms that must be regulated under the conditions of potassium shortage. We show that the activation of the proton pump, Pma1, and the activation of the bicarbonate reaction sequence are the regulators of potassium homeostasis. We also show that potassium homeostasis is an example of non-perfect adaptation: The intracellular potassium concentration depends on the external potassium concentration and is only regulated to keep minimal levels of potassium required for survival. This is different from other homeostatic systems such as osmoregulation [20], where certain stationary systems characteristics perfectly adapt, irrespective of the external conditions.

## Results

### Potassium starvation experiments

**Figure 1:**
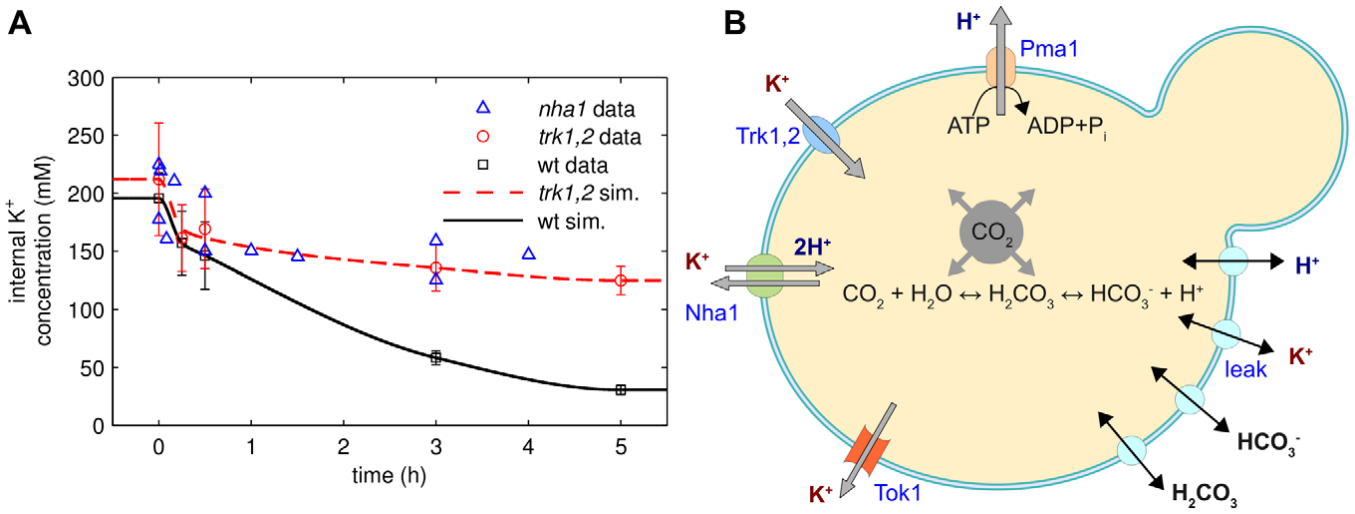
Homeostatic response to potassium starvation. (A) Experimental time courses of internal potassium concentrations in wild-type (WT) cells, *trk1,2* double mutants and *nha1* mutants (symbols). Cells were grown in 50 mM KCl and resuspended in Translucent 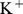-free medium at t = 0. Solid lines (“sim”) are fits to the model using the reverse tracking approach (see text). (B) The components of the minimal biophysical model.

To study the response of *S.c.* cells to an abrupt decrease of external potassium, we performed potassium starvation experiments using 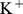 and 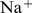 free media. Cells grown in non-limiting potassium (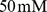 KCl) were washed with 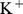-free YNB medium (YNB without amino acids and ammonium sulphate, Formedium UK, CYN7505 plus 2% glucose, traces of KCl left: 15 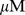, hereafter referred to as Translucent 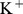-free medium [21]) and resuspended in the same medium [12]. The time course for changes in intracellular potassium concentrations for the wild type strain exhibits two different phases (Figure 1A). In the first hour of starvation there is a large net efflux of potassium indicated by the rapid decrease in the intracellular concentration. Loss of potassium slows down in the second phase and the internal concentration slowly approaches a new stationary state (Table 1). Although the cells cannot perfectly adapt to the large concentration gradients they are able to keep a certain amount of potassium required for survival (approx. 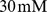). Interestingly, the second phase of potassium loss is slower for the *trk1,2* double mutant than for the wild type (wt). This is surprising, because it is believed [3] that increased uptake of potassium via Trk1 even at very low external potassium concentrations is a major mechanism of potassium homeostasis. Thus, one would have expected the concentration of internal potassium in the *trk1,2* mutant to be lower than in the wild type. The time course for the *nha1* mutant is not significantly different from the *trk1,2* mutant (see also Figure S7 in Text S1).

**Table 1:** Optical densities during starvation.

Strain	Time of starvation (hours)				
	1	2	3	4	5
wt	0.3	0.39	0.45	0.5	0.5
trk1,2	0.3	0.35	0.4	0.42	0.43

Optical densities for the wild type and the *trk1,2* double mutant corresponding to the potassium starvation experiments of Figure 1A.

### A mathematical model for potassium transport

Multiple signaling pathways modulate the activity of the various transport systems involved in potassium homeostasis [2]–[6], [14], [15], [22]–[24]. However, it is not entirely clear which of these signals are essential to achieve homeostasis and how they are acting under the constraints set by the thermodynamics of ion transport. To study these constraints, we developed a minimalistic mathematical model which incorporates the essential parts known to be important for potassium homeostasis. The model describes the dynamic coupling between the intracellular potassium concentration 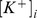, internal pH (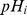), carbon dioxide concentration 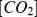, membrane voltage 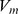, and cell volume 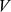. A complete description of the equations and parameter values is given in the Materials and Methods section and derivations can be found in the Text S1. Here, only the basic model structure is given:
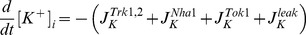
(1)

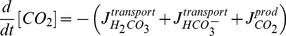
(2)

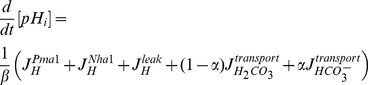
(3)

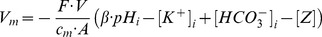
(4)

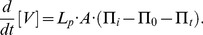
(5)Equation (1) links the temporal change of the intracellular potassium concentration to the various potassium transport fluxes 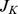 (Figure 1B). The model comprises the Trk1,2 system (abstracted as a single system, 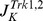), the Nha1 antiporter (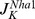), and the Tok1 channel (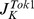). To mimic the joint contribution of other, mainly non-specific transport pathways for potassium (e.g. Nsc1) we added a potassium leak current 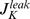 to the model. The Ena1 ATPase is neglected because it is known to be inactive at the relatively low external pH used in the experiments [15].

The dynamics of carbon dioxide (Equation (2)) is coupled to the transport fluxes of bicarbonate 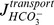 and carbonic acid 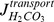. These transport rates are given in the Materials and Methods section (Equations (18–19)) and a detailed derivation of the bicarbonate model [25] is given in the Text S1. Carbon dioxide is produced in various metabolic processes such as the TCA cycle or pyruvate decarboxylation. It is impossible to model all these processes explicitly, but we incorporate them in the effective metabolic carbon dioxide production flux 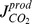. This flux is an input to the model and was initially assumed to be constant.

The change in pH (Equation (3)) per change in proton concentration is described by the buffering capacity 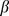. In principle, 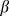 is a function of the internal pH, but due to the combined action of various buffering species [26] it can be approximated by a constant for a wide range of intracellular pH values. In addition to the proton fluxes via the 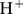-ATPase Pma1 (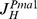) and the Nha1 antiporter (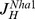) there are many other proton transport pathways in yeast. The corresponding net flux is subsumed in the proton leak current 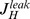. The effective proton flux originating from the bicarbonate reaction sequence is given by the term 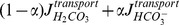, where 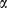 is the pH-dependent fraction of dissociated carbon dioxide.

The membrane potential (Equation (4)) is modeled as a charge balance equation (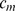, specific membrane capacitance; 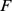, Faraday constant; 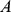, surface area of the cell) [27]. We explicitly modeled the charges carried by potassium, total protons (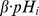) and bicarbonate. The remaining net charges contributing to the membrane potential are subsumed in 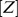, which is determined by the initial conditions of the dynamic variables in the model.

The cell volume (Equation (5)) depends on the balance between internal osmotic pressure 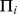, external osmotic pressure 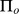 and turgor pressure 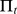 [28]. Ion transport processes change the intra- and extracellular solute concentrations and thus have an osmotic effect (Equations (24–26)) in Materials and Methods). The resistance against volume changes is given by the hydraulic permeability parameter 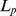 [29].

The concentration and voltage dependent kinetics of all transport systems were described by simple thermodynamic consistent relationships. The driving force for the transport fluxes of ions across the plasma membrane can be written as the difference 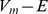 of the membrane potential and the equilibrium potential 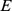. The equilibrium potential depends on the concentrations and stoichiometry of the ions transported, see Equations (12–14) in the Materials and Methods section. For the potassium fluxes in Equation (1) and the proton leak in Equation (3) we assumed linear relations (Ohm's law) of the form 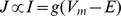 between the driving force and the transport flux 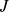, or the corresponding electrical current 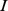, respectively. For the leak currents 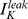 and 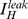 we initially assumed constant conductivity parameters 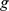 (Equations (9) and (11) in Materials and Methods). The conductivity of the transport proteins Trk1,2, Nha1 and Tok1 was modeled as a function of the membrane voltage, see Equations (6–8) in Materials and Methods.

This minimalistic model captures the essential biophysical and thermodynamic constraints under which control of potassium homeostasis operates. Despite the simplicity of the model, the experimental data was not sufficient to uniquely identify all the parameters. We decided to use this flexibility to explore the parameter space for regions that are consistent with the data and performed extensive parameter scans and sensitivity analysis simulations. However, we were unable to identify a single parameter combination which reproduced the experimental time courses for the wild type strain observed in Figure 1A. In the model, all potassium inside the cell was rapidly and completely lost upon starvation (Figure S4A in Text S1). Based on our model simulations, this believed to be caused by a strong efflux via the Nha1 antiporter driven by the large concentration gradient across the plasma membrane. This model behavior is robust against various model variations, including the incorporation of an intracellular potassium storage mechanism that mimics the contribution of intracellular compartments to potassium retention. Thus, we conclude that further dynamic mechanisms counteracting the strong potassium gradient are essential for homeostasis. Importantly, the model described so far incorporates only the biophysics of transport but does not account for gene regulatory, signal transduction or metabolic events affecting the transporter activity.

### Predicting the regulators with the reverse tracking algorithm

The fact that the minimal model is not able to reproduce the experimental time courses for potassium starvation means that there are some unmodeled dynamics that are not captured by the model. Under the working hypothesis that the model covers the major biophysical effects of potassium transport we assumed that there are additional regulatory responses to a shortage of potassium. Available knowledge [2], [3] and data is currently not sufficient to develop exhaustive models for the metabolic, signal transduction and gene regulatory responses to potassium starvation. It is not even clear which of the transporters or other components are activated or deactivated for the maintenance of homeostasis. In engineering terms [30], neither the regulators nor the signals triggering their action are sufficiently characterized.

**Figure 2:**
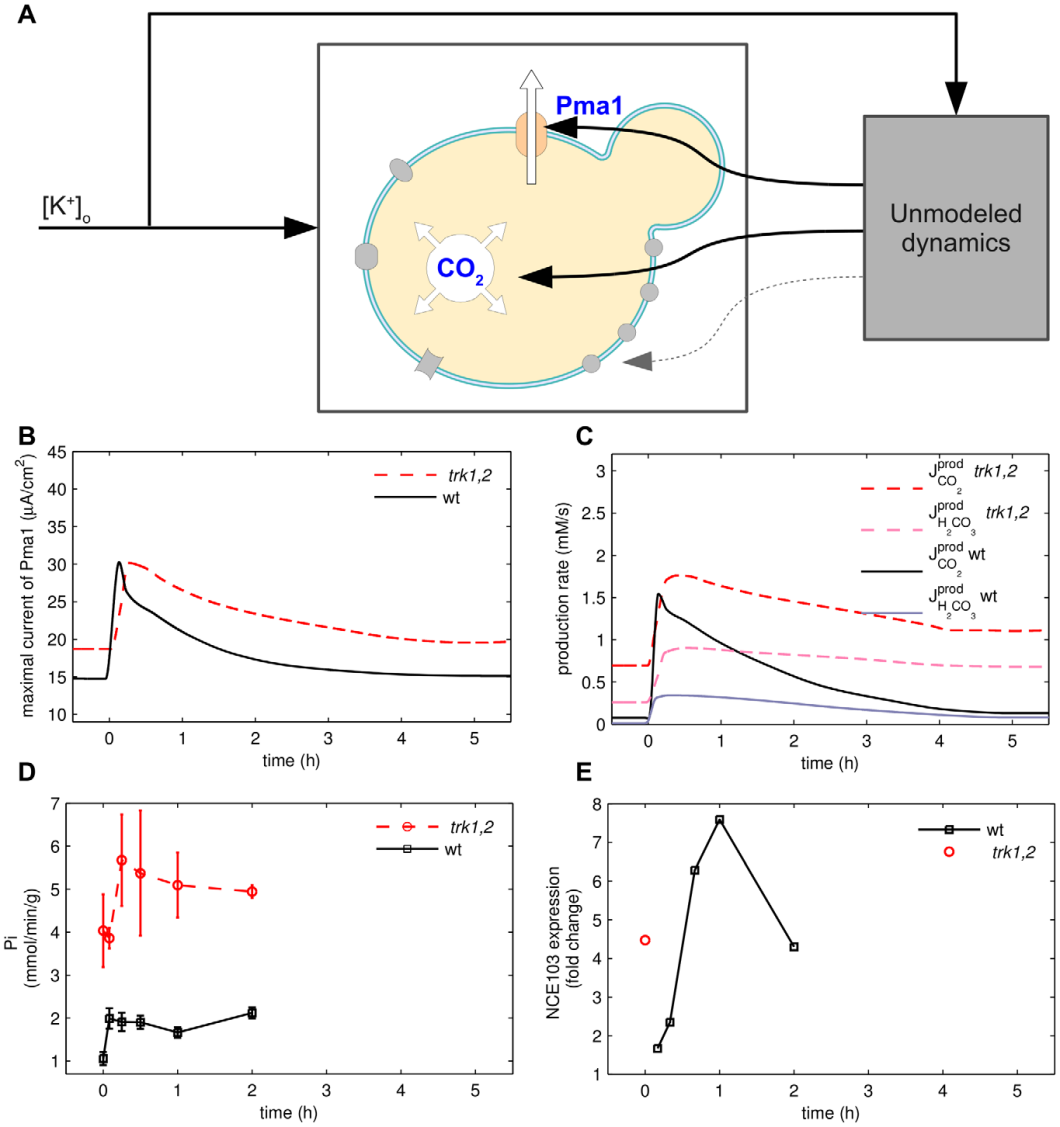
Regulation of potassium starvation. (A) The tracking approach to detect potential regulators of homeostasis. Parameters which are constant in the minimal model are now considered as input functions. A parameter is called a potential regulator if it can be chosen to recover (“track”) the experimental time courses. (B,C) The predicted activity changes for Pma1 (B) and the bicarbonate reaction system (C) in response to potassium starvation. (D) Time course of ATPase activity for Pma1. (E) Time course of gene expression for the *NCE103* gene encoding carbonic anhydrase in the wild type strain. Confirmatory qRT-PCR measurements yield a 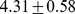 fold increase of the mRNA level in the wild type after 60 minutes of potassium starvation. For comparison, the expression in non-starved *trk1,2* double mutant with respect to the wild type strain is depicted. The mRNA levels for *NCE103* in *trk1,2* double mutants growing at 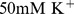 are higher by a factor of 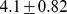 compared to the wild type strain (qRT-PCR measurements).

To overcome this limitation, we combined our minimal biophysical model with an inference algorithm for unmodeled dynamics. We assumed that the unknown regulatory events modulate the activity of the transport systems or other components in the model. Mathematically this means that a constant parameter in the model might in fact not be constant, but a function of time. For example, the maximum conductivity 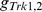 (see Equation 6) of the Trk1,2 transport system could be influenced by signal transduction events [3], [31] in response to low potassium. Any attempt to explicitly model this regulation by additional equations is hindered by insufficient knowledge of the structure and dynamics of the regulatory networks involved. However, one might recoin the question and ask: “Is there a function 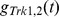 such that the given experimental time course 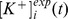 of intracellular potassium and the time course 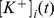 predicted by the model are in sufficient agreement?”. If such a function 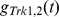 would exist we would regard the modulation of the Trk1,2 transporter as one potential regulatory mechanism and Trk1,2 as a *potential* regulator of potassium homeostasis. However, there might be another parameter 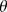 (e.g. 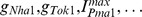) associated with a transporter or another component in the model for which a time course 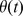 exists such that experimental data can be reproduced. Our strategy was now to test different parameters and corresponding processes for being potential regulators, see Figure 2A. We define a transporter or any other component in the model to be a potential regulator if a tracking control signal 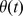 exists which changes the activity of the component in such a way that the experimental time course and stationary data can be reproduced. We refer to this inference approach as the reverse tracking algorithm, a more detailed mathematical explanation is given in Materials and Methods.

We used the reverse tracking algorithm to test the transporters Trk1,2, Ena1, Nha1, Tok1 and Pma1 and the activity of the bicarbonate reaction for being potential regulators of potassium homeostasis and then compared the predicted tracking control signals to experimental observations. There is no tracking control signal for the major uptake system Trk1,2; see Figure S1 in the Text S1. This is in contrast to the prevailing view that increased uptake of potassium via Trk1 is essential for potassium homeostasis under starvation conditions. The loss of potassium after starvation is slower in *trk1,2* double mutants (see Figure 1A) than in wild type cells. It was experimentally observed [12] that these double mutants have a more negative membrane potential than wild type cells under starvation conditions and also when external potassium is plentiful. This stronger membrane potential (see also Figure S3 in the Text S1) counteracts the outwardly directed potassium concentration gradient and thus explains the higher potassium levels after starvation. Taken together, these results show that the uptake of potassium via Trk1,2 is not the primary mechanism to prevent excessive loss of potassium under starvation conditions.

Although we found a tracking control signal for the Nha1 antiporter, we excluded it from our list of potential regulators based on two observations. First, as indicated in Figure 1A, the time course of potassium loss in *nha1* mutants is slower than in the wild type and similar to the *trk1,2* mutants. Secondly, it was demonstrated that the influence of Nha1 on the internal potassium concentrations decreases with time [11]. This is in contradiction to our predicted tracking signal (Figure S2 in Text S1), which is nonmonotonic in time.

Similarly, the unspecific transport pathways (leak currents) were excluded, because it is not plausible that unspecific transporters are regulated for the specific purpose of potassium homeostasis. This is based on the well founded assumption that all potassium specific transporters are active under our experimental conditions are known [3] and included in the model. The non-specific cation uptake system NSC1 can be excluded, because our medium contains enough calcium to render NSC1 inactive [32]. The proton flux 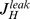 includes many co-transport mechanisms with nutrients and other molecules. It is thus unlikely, that one of these transport mechanisms is specifically regulated in response to potassium starvation.

The remaining parts in our model are the Pma1 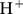-ATPase and the bicarbonate reaction sequence. For both of them, the reverse tracking approach predicts a rapid burst of activity in response to the rapid removal of external potassium (Figure 2B and 2C). Activation of proton pumping by Pma1 (Figure 2B) hyperpolarizes the plasma membrane, which counteracts the large concentration gradient of potassium and thus limits potassium efflux. An increased reaction flux (see Figure 2C) through the bicarbonate system has a similar effect: The negative charges carried by bicarbonate increase the magnitude of the membrane potential and thereby compensate the potassium gradient.

### Experimental validation of the predicted regulators

**Figure 3:**
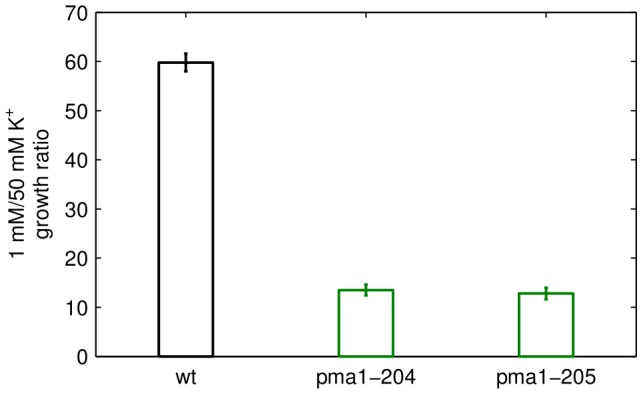
PMA1 mutants with decreased expression and ATPase activity. Strains RS514 (wild type, WT), RS515 (*pma1–204*) and RS516 (*pma1–205*) were grown in YNB-based medium (supplemented with adenine and histidine) with 2% galactose to maintain Pma1 activity from plasmid pYCp50-GALp::PMA1. Cells were diluted to an OD600 of 0.04 in Translucent 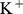-free medium (plus with 2% glucose) containing 1 mM or 50 mM KCl. Growth was monitored for 17 h. Data represent the growth ratio at 1 and 50 mM KCl and are mean 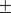 SEM from 3 determinations.

To test the prediction that Pma1 is activated after potassium starvation, we measured Pma1 activity from crude membrane preparations [33] using an in vitro method that has been extensively established as a faithful measure of in vivo Pma1 function [6], [33], [34]. Indeed, the activity measurements confirm the prediction of the reverse tracking algorithm that Pma1 activity increases rapidly (timescale of 10 minutes) and slowly declines during the first hours of potassium starvation (Figure 2D). Control experiments revealed that Pma1 protein levels do not change under these conditions. Moreover, we also observe, as predicted by the model, that the Pma1 activity is higher in the *trk1,2* mutant than in the wild type strain throughout the time course of potassium starvation (Figures 2B and D). To further substantiate that the activation of Pma1 is essential for the response to low potassium, we measured growth for Pma1 mutants *pma1–204* and *pma1–205* [35] with decreased expression and ATPase activity (33 and 50% of wild type). Figure 3 shows that the ratio of the growth rates at 1 mM and 50 mM external potassium is much lower for the mutant strains than that of the wild type. These results are in line with the recent finding that the *brp1* mutant, which is a *PMA1* promotor deletion, that leads to decreased Pma1 protein levels, presents markedly decreased growth in low potassium medium and defective rubidium uptake [36].

The second prediction from the reverse tracking approach was an increased reaction flux for the bicarbonate system (Figure 2C). This prediction is supported experimentally by an increased mRNA expression of the *NCE103* gene coding for carbonic anhydrase, the enzyme catalyzing the bicarbonate reaction (Figure 2E). This result was part of a genome-wide transcriptomic analysis, using DNA microarrays, of the response to potassium starvation (0–120 min) to be published elsewhere (Barreto et al., submitted). It was shown earlier that protein and mRNA levels of carbonic anhydrase are highly correlated [37]. A qRT-PCR measurement confirmed the increase in *NCE103* expression in wild type cells shifted to 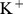 free medium. After 60 minutes of potassium starvation, the *NCE103* mRNA levels increase more than four-fold (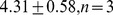 independent experiments). These results show that activation of both Pma1 and the bicarbonate reaction sequence are essential for the control of internal potassium concentrations. In non-starved cells the expression of *NCE103* is higher for the *trk1,2* double mutant than for the wild type (single dot in Figure 2E). A confirmatory semi-quantitative RT-PCR measurement using the same RNA sample as in the microarray experiment and one RNA sample from independent cultures yielded a mean expression ratio of 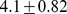 (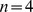 data points) for *trk1,2* relative to the wild type. These results suggest a simple explanation for the reported hyperpolarization of the *trk1,2* double mutant [12]: A high activity of the bicarbonate reaction sequence means that many protons and many bicarbonate ions are produced. Together with a more active proton pump (Figures 2B and D), this results in a more negative membrane potential that counteracts the outwardly directed potassium gradient. The consequence is a higher intracellular concentration of potassium (Figure 1A) in *trk1,2* double mutants than wild type cells.

### Non-perfect adaptation to external potassium concentrations

Homeostatic control of a cellular function in response to a changing environment is often mediated by a negative feedback loop. A change in the input signal (e.g. the external potassium concentration) is counteracted by this feedback loop in order to keep an essential cellular quantity (e.g. the intracellular potassium concentration) in a range sufficient for the cell's function. One particular type of feedback is integral control, where the control signal is the time integral of the difference between the reference and the actual quantity [30]. Integral control was observed for a number of cellular processes including bacterial chemotaxis [38], [39] and osmoregulation [20]. A characteristic property of integral control is perfect adaptation, where the steady state input is independent of the steady state output. For potassium this would mean, that the same intracellular potassium concentration (output) is approached irrespective of the extracellular potassium concentration (input).

**Figure 4:**
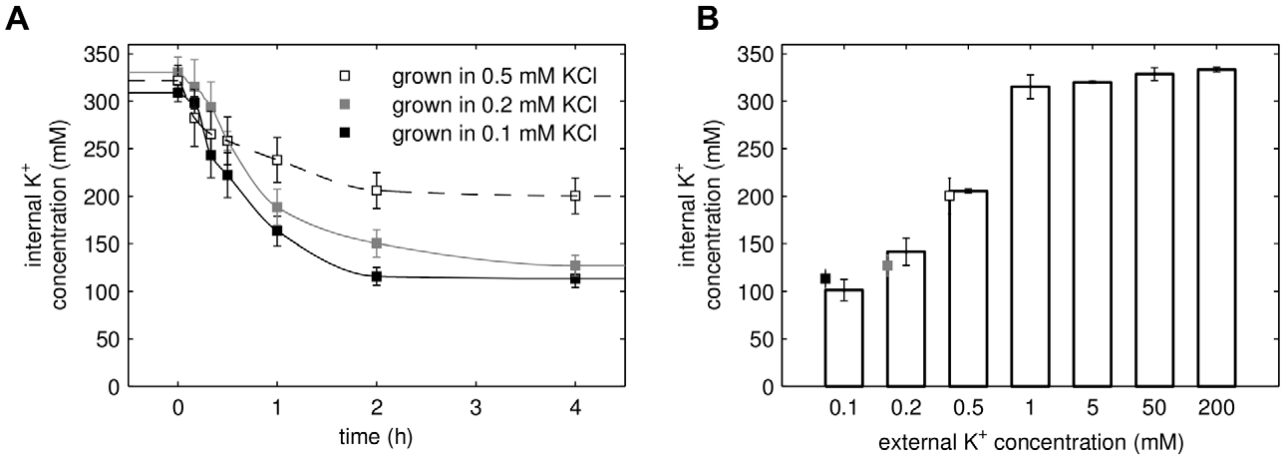
Relationship of external and internal potassium. (A) Cells grown in 50 mM KCl were resuspended in 0.1, 0.2 and 0.5 mM KCl and the time course of internal potassium was monitored. The lines show the data fit obtained from the reverse tracking algorithm. (B) Internal potassium concentration in cells grown overnight at different external potassium concentrations. The steady state concentrations from (A) are indicated as squares.

The activation of proton transport by Pma1 and the activation of the bicarbonate system counteracting low external potassium indicate the existence of a negative feedback loop. To further investigate this feedback, we have modified the potassium starvation experiment. As before, cells were grown at 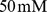 external potassium, but now resuspended in media with different external potassium concentrations. The potassium efflux and the stationary internal concentrations are different for the different external concentrations, which is also reflected by the model (Figure 4A). To test whether these stationary intracellular concentrations are characteristic for the external potassium, we grew cells overnight in media with different external potassium concentrations (Figure 4B). When external potassium is plentiful (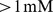), the internal concentration attains an upper limit of approx. 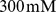. For low external potassium (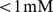), the internal concentration is proportional to the external and agrees with the stationary states of Figure 4A. These experiments show, that perfect adaptation by integral control is not a characteristic of potassium homeostasis for low external potassium. The molecular function and characteristics of this feedback have to be further explored.

## Discussion

**Figure 5:**
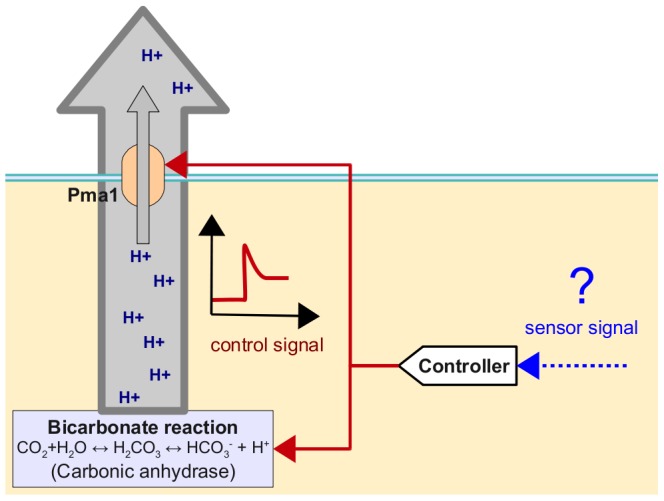
Proposed mechanism of potassium homeostasis. Changes of the external potassium concentration are sensed by an unidentified sensor system either directly or indirectly, e.g, via the membrane potential, internal potassium or pH changes. The sensor signal triggers a modulation of proton fluxes using the bicarbonate reaction system and the Pma1 proton pump as regulators.

In summary, we found that direct regulation of potassium transport proteins is not sufficient for the maintenance of viable potassium levels inside the cell. Although the presence of Trk1,2 influences the dynamics of potassium loss under conditions of low potassium, the regulation of their activity is not the main regulatory process. Cells lacking these proteins have higher intracellular potassium concentrations and the loss of potassium after a rapid shift to low external potassium is slower than in wild type cells. The adaptation to low potassium requires a rapid modulation of proton fluxes as a rescue operation via the increased production of bicarbonate and the activation of the 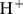-ATPase Pma1 (Figure 5). The observation that the internal steady state potassium concentration is determined by the external concentration indicates, that potassium homeostasis is an example of non-perfect adaptation, excluding the existence of integral control. The detailed sensing and signaling mechanisms remain to be elucidated and currently we cannot distinguish whether changes in internal or external potassium are sensed directly or indirectly, e.g., as changes of the membrane potential.

Although we cannot completely rule out the possibility that other transport systems not considered in the model contribute to homeostasis, we have reason to believe that our model covers the dominant effects required for the maintenance of viable potassium levels under starvation conditions. All experiments were performed in the presence of calcium, which renders the activity of the calcium blocked non-selective cation pathway Nsc1 unlikely. In addition, non-specific transport of potassium is covered in the model by the leak current. The information about potassium storage in intracellular compartments in the literature is limited. To test the influence of intracellular potassium fluxes originating from an intracellular storage mechanism, we added a hypothetical compartment which can release potassium in response to starvation. This modification did not change the qualitative behavior of the model and was not sufficient to explain the slow efflux of potassium and the maintenance of sufficient intracellular potassium after starvation. Thus, we excluded this modification from the model.

Many cation transporters are evolutionarily conserved in other yeast species and even in higher plants [1]–[3]. However, the current knowledge for these organisms is not as detailed. Considering the importance of ion homeostasis for some pathogenic yeasts [40] and for the growth and development of plants, the question of whether the regulation of proton fluxes plays a similar dominant role as in *S.c.* is an interesting starting point for future research.

The development of dynamic mathematical models requires a compilation of all parts and processes which could potentially be important for a cellular mechanism under consideration. Other processes believed to be negligible are often lumped together in the parameter values of the model. The decision of which processes to incorporate or to neglect is often hampered by insufficient biological knowledge. Incorporating too many details is impractical and leads to overly complex models with many parameters and little predictive power. On the other extreme are simplistic models which potentially neglect important processes and cannot reproduce the experimental data. We believe that our strategy to start with such a minimal model and to infer unmodeled dynamics with a reverse tracking approach might be of broader interest in systems biology. The reverse tracking algorithm provides (i) candidate points of applications for regulatory signals not explicitly captured by the model and (ii) an estimate of the corresponding time dependent regulatory signal. We emphasize that these potential regulatory signals have to be checked for biological plausibility and have to be validated by experiments. It can be applied when the core model for the process of interest is “underfitted”, i.e. when it can not sufficiently reproduce the experimental data because other regulatory process influence the parameters in the model. Its main advantage is that it can be applied even when an explicit modeling of the processes generating these regulatory inputs is beyond reach. On the other hand, the algorithm can be used as a tool for prioritizing experiments. In combination with experiments, it also may also help to indicate which model extensions are most promising.

## Materials and Methods

### Mathematical model

The basic structure of the mathematical model is given by Equations (1–5) in the Results section. Here we report the details of the kinetic relationships. Parameter values, initial conditions and derivations are provided in the Text S1. In the following 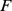, 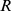 and 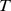 denote the Faraday constant, the gas constant and the temperature.

#### Passive and secondary active potassium and proton transport

The concentration and voltage dependent transport currents of the passive or secondary active potassium and proton transporters (Equations (6–11) below) are described by the Ohmian relation 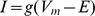, where the conductivity 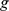 is either constant (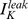 and 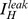) or a function of the membrane voltage (Trk1,2, Nha1, Tok1). The voltage dependent conductivity of the transporter 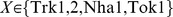 was described by the function
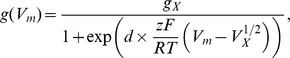
which can be derived from a simple model for the stochastic opening and closing of a transporter or channel [27], [29], [41]. The parameters 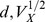 and 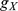 for Tok1 were taken from the literature [42]; for Trk1,2 we estimated them from electrophysiological data [43]; see Figure S6 in Text S1. For Nha1 we assumed this voltage-dependent conductivity on the basis of [44]. This approach leads to the following transport kinetics (here 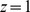)
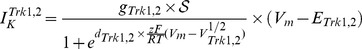
(6)

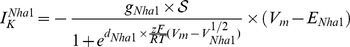
(7)

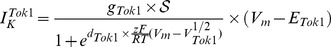
(8)

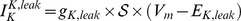
(9)

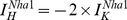
(10)

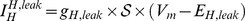
(11)with the equilibrium potentials
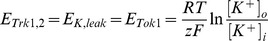
(12)

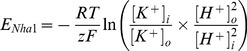
(13)

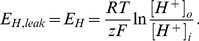
(14)The electric currents 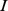 are related to the mass fluxes 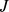 by
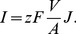
(15)and the factor 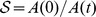 was introduced to correctly incorporate the dependence of the conductance parameters on the surface area 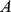 of the cell.

The transport current for Pma1 depends on the free energy 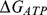 of ATP hydrolysis and was modeled as [45]

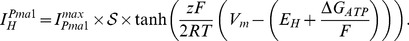
(16)


#### Bicarbonate reaction

The model in [25] for the bicarbonate reaction sequence was supplemented by an effective metabolic carbon dioxide production or consumption flux 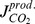. The production rate 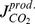 is an input to the model. Note that the production changes with the volume even if the amount of produced CO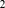 does not change. This is taken into account by the relationship
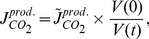
(17)where 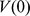 denotes the initial volume and 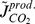 is the volume independent rate, which is input to the model.

The dynamics of CO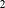 in Equation (2) depends also on the transport fluxes for carbonic acid and bicarbonate
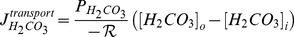
(18)

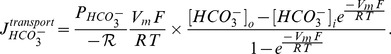
(19)The parameters 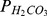 and 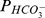 are permeabilities for carbonic acid and bicarbonate respectively, and 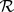 is the volume to surface ratio of the cell, see Text S1. The assumption behind Equation (18) is that the flux of carbonic acid is proportional to its concentration gradient. Equation (19) is a Goldman-Hodgkin-Katz flux equation for the electrodiffusive transport of ions across the membrane [25], [41].

The fraction of total undissociated carbon dioxide
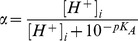
(20)(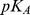 of carbonic anhydrase) enters the rate of proton production in Equation (3), see Text S1 for a detailed derivation. The production fluxes of carbonic acid and bicarbonate
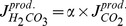
(21)

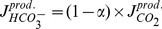
(22)depend on 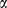 and on the production rate of carbon dioxide 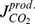.

#### Cell volume

For the dynamics of the cell volume (Equation (5)) we used a model from the literature [28] which is based on the balance of internal and external osmotic pressure (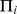 and 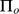) and turgor pressure (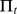):
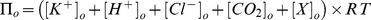
(23)

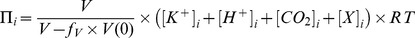
(24)

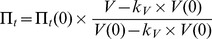
(25)

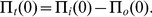
(26)Here, 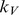 is the fraction of the non-osmotic cell volume and 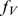 determines the elasticity of the cell wall. The internal and external concentration 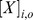 of other osmotically active substances are constant (see Text S1). A cell volume simulation for wildtype and *trk1,2* mutant can be found in Figure S5 in Text S1.

#### Potassium starvation

The external potassium concentration 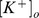 is an input to the model. The shift from a medium containing 50 mM KCl to the starvation medium was described by
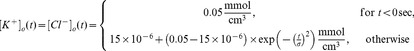
(27)with 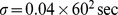.

### Reverse tracking algorithm

Equations (1–5) have the form of a differential algebraic control system
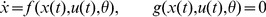
(28)with 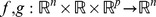. Here, 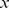 denotes the dynamical variables (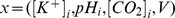) and Equation (4) for the membrane potential corresponds to the algebraic equation 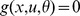. The scalar input function 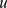 is given by the external potassium concentration 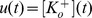. The solution of this system for given values of the parameters 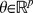 and a given input function 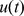 is denoted by 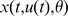. Assume now, that we can observe 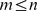 of the components 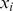 of 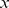 experimentally. We collect the experimentally observable components in 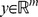. This can be written as 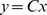 with a 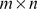 matrix 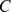 with binary elements 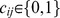. For an experimentally observable variable 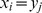 the 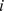-th column of 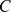 has a single entry 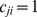 and 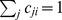. A zero column with 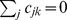 indicates that 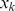 can not experimentally be observed and is thus excluded from 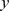.

Assume further, that we have experimental data 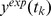 for certain time points 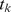 in response to the known input function 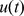. Most parameter estimation techniques aim to minimize the squared error
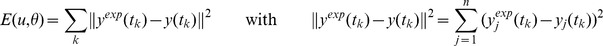
over the parameter vector 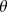 in order to bring the model prediction 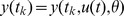 for a given input 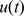 close to the experimental data 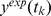. However, it might be the case that the minimum error 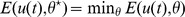 is still too large so that the model cannot be regarded as a reasonable description of the data. This could mean that a dynamical process not explicitly accounted for renders at least one component 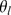 of the parameter vector 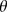 to be a time dependent function instead of being constant. The reverse tracking algorithm aims (i) to identify, which of the components 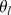 of 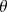 are *potentially* time dependent and (ii) to predict the time course 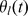 which minimizes the error. Although the unmodeled dynamical process might effect more than one component, we consider for simplicity each component 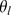 separately and solve the problem
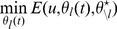
(29)

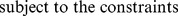


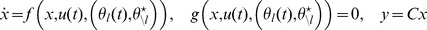
for each component 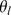 separately. Here, 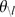 denotes the parameter vector 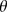 with the l-th component excluded. We then regard 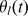 as a potential regulatory input, if the problem (29) has a solution with a minimum error smaller than a predefined threshold 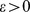: 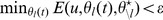. There might be more than one potential regulatory input 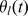 and the decision of which of these are real can only be made from biological considerations or from additional validation experiments. For example, it might be that 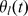 has a huge magnitude or takes unrealistic values which could be used to exclude 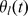 from the list of potential regulatory inputs.

Mathematically, problem (29) is an optimal tracking problem, which often can be solved by a feedback control law [46]. This means that the function 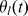 is updated according to the local error 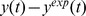 at time 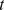. For a scalar 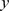 we found the integral controller [30]

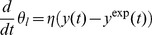
(30)to be a good solution. During a reverse tracking run, this equation is numerically integrated in parallel with the dynamic equations (28). Here, 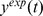 is a least squares spline fit to the experimental data points 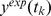. Details and suitable parameter values for 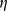 are provided in the Text S1.

### Strains and medium

Details about the wildtype strain BY4741, the related *trk1,2* mutant and the Translucent 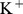 free medium can be found in [12], [21].

### Potassium starvation experiments and concentration measurements

Cells were grown in Translucent 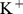-free medium supplemented with the indicated amount of KCl to an OD600 of 0.4–0.6. Intracellular potassium concentrations were measured by atomic emission spectrometry after extracting the cells with acid as previously described [12]. The time course of internal potassium was obtained by growing the cells in 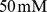 KCl, then cells were washed with Translucent 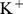-free medium (traces of KCl left: 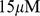) and resuspended to the same 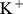 free medium or containing the indicated KCl concentrations. Apart from the washing procedure the medium contains 2% glucose.

### Gene expression measurements for *NCE103* coding for carbonic anhydrase

Data for *NCE103* expression changes upon potassium starvation was obtained in the context of a genome-wide transcriptomic analysis by DNA microarray (Barreto *et al.*, Manuscript submitted). Microarray data has been deposited at NCBI's Gene Expression Omnibus [47] and are accessible through GEO Series accession numbers GSE24711 (trk1 trk2 data) and GSE24712 (time-course data). Briefly, wild-type strain BY4741 cells were grown in Translucent medium supplemented with 50 mM KCl to OD 0.8. Cells were centrifuged and resuspended either in fresh Translucent medium with 50 mM KCl or without potassium. Samples (20 ml) were taken at 10, 20, 40, 60 and 120 min by rapid filtration from 4 biological replicates. Total RNA was extracted by using the Ribo PureTM Yeast kit (Ambion) following the manufacturers instructions. cDNA was prepared and indirectly labeled with Cy3 and Cy5. Images with a resolution of 10 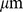 were analyzed with the GenePix Pro 6.0 software (Molecular Devices).

Microarray data was confirmed by qRT-PCR using independent RNA samples. To this end, 60 ng of RNA were amplified using oligonucleotides RT_ *NCE103*_5 (TCATTACCTGTCGCACTG) and RT_ *NCE103*_3 (CACAAAAGTTACCCCAAAA) and the QuantiTect SYBR Green PCR Kit (Quiagen).

### Membrane isolation and determination of Pma1 activity

Cell cultures were grown at 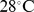 in Translucent YNB medium containing 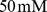 KCl to OD660 0.6, then washed with Translucent 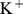
- free medium and resuspended in the same medium without KCl. At the indicated times, cell samples were pelleted by centrifugation, resuspended in 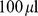 of fresh media (with KCl for t = 0 and without KCl for the remaining samples), incubated for 5 minutes and frozen in liquid nitrogen. For the crude membrane purification, 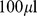 of 3× extraction buffer (0.3 M Tris-HCl pH 8.0, 180 mM KCl, 30 mM EDTA, 6 mM DTT and Protease Inhibitor Cocktail (Roche)) was added to the thawed samples and cells were broken by vortexing in the presence of an equal volume of glass beads. 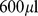 of GTED20 buffer (20% glycerol, 10 mM Tris-HCl pH 7.6, 1 mM EDTA and 1 mM DTT) were added to the crude extract, which was then centrifuged 5 minutes at 2000 rpm. The supernatant was transferred to a new tube and centrifuged 20 minutes at 13000 rpm. The insoluble fraction was resuspended and homogenized in 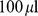 of GTED20. The total amount of protein present was estimated using the Bradford assay (BioRad). The amount of Pma1 present in this protein fraction was estimated by comparing the amount of Pma1 to a protein standard curve separated in SDS-PAGE gels stained with Coomassie Blue. In a microtiter plate, 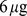 of total protein (which corresponds to 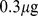 of Pma1) were assayed in the presence and absence of a Pma1-specific inhibitor, dietilstylbestrol (DES, final concentration 0.2 mM). The reaction was started by adding 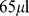 of the reaction buffer (50 mM MES-Tris pH 5.7, 5 mM MgSO4, 50 mM 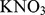, 5 mM Na Azide, 0.3 mM Molybdate, 2 mM ATP) and the plate was incubated for 20 minutes at 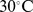. The reaction was stopped by adding 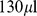 of detection solution (2% sulphuric acid, 0.5% ammonium molybdate, 0.5% SDS, 0.1% ascorbic acid) and the color was allowed to develop for 5 minutes before reading the absorbance in microplate reader (BioRad) at 750 nm. Residual activity values in the presence of DES were subtracted from the absolute activity values to obtain the Pma1 activity measurements. The results represent the average of at least 4 measurements at each time point and essentially identical results were observed in two separate experiments. Measurements of Pma1 activity are expressed in mmol/min/g Pma1. Error bars represent the standard deviation.

## Supporting Information

Text S1 Supporting text containing additional information about the mathematical model.
(PDF)
