# Supplementary material for: Potassium Starvation in Yeast: Mechanisms of Homeostasis Revealed by Mathematical Modeling
Source: PLoS Comput Biol. 2012 Jun 21;8(6):e1002548. doi: 10.1371/journal.pcbi.1002548 (PMC3380843; doi:10.1371/journal.pcbi.1002548)
Supplement: Text S1 — Supporting text containing additional information about the mathematical model. (PDF) [file pcbi.1002548.s001.pdf]

Text S1 for: "Potassium starvation in yeast: Mechanisms of homeostasis revealed by mathematical modeling."

Matthias Kahm[1], Clara Navarrete[2], Vicent Llopis-Torregrosa[3], Rito Herrera[2], Lina Barreto[4], Lynne Yenush[3], Joaquin Ariño[4], José Ramos[2], Maik Kschischo[1,*]
1 Department of Mathematics and Technology, University of Applied Sciences Koblenz, RheinAhrCampus Remagen, Südallee 2, 53424 Remagen, Germany
2 Department of Microbiology, Campus de Rabanales, University of Córdoba, E-14071 Córdoba, Spain
3 Instituto de Biologia Molecular y Celular de Plantas UPV-CSIC, Ciudad Politécnica de la Innovación, Universidad Politécnica de Valencia
4 Institut de Biotecnologia I Biomedicina and Department of Biochemistry and Molecular Biology, Universitat Autònoma Barcelona, Cerdanyola del Vallès, 08193 Barcelona, Spain
∗ E-mail: kschischo@rheinahrcampus.de

1 Mathematical Model

The model describes ion transporters and channels as resistors, the membrane as a capacitor and the proton pump as a battery in an electric circuit [1–5]. Transport of ions across the plasma membrane changes their respective intracellular concentrations, the volume and the membrane voltage. Apart from the H^+-ATPase Pma1, the transport systems were modeled according to Ohm's law using either constant or voltage dependent conductivities. In the latter case, the conductivity as a function of voltage is given by the maximum conductivity times the probability for the transporter to be active. This probability can be derived from a two state voltage gated model for the activity of the transporter [1].

The core model comprises the transport systems Trk1,2, Pma1, Nha1, Tok1 as well as potassium and proton leakage currents. We used information from the literature to model the voltage and concentration dependent kinetics of these transport systems. For Trk1 and Trk2 we used electro-physiological measurements [6] to fit a two state model as described above; see Section 2.6. Both proteins were subsumed as one transport system since there is no data for the individual transporters. In addition, the low affinity transporter Trk2 is considered to be less relevant. For Tok1 we used a model from the literature [7]. For the potassium leak we assumed a constant conductivity and used published data [6] (Figure 9c). Measurements in *Neurospora crassa* were used for the constant conductivity of the proton leak [8]. The proton pump Pma1 was described by a simple thermodynamic approach [9] using values for the maximum pump current from electro-physiological measurements [10].

For the antiporter Nha1 there is currently no electro-physiological data available. We decided to describe Nha1 as a voltage gated channel, see Section 2.4 for details. The membrane potential is modeled according to a capacitor equation, which satisfies the requirements of the cell's electro-neutrality. For the dynamics of the cell volume we used a model from the literature [11] which is based on the balance of internal (Π_i), external (Π_o) and turgor pressure (Π_t).

1.1 Units, conventions and variables

Physical units:

- V: Volt
- A: Ampere
- S: Siemens
- J: Joule

- F: Farad
- K: Kelvin
- C: Coulomb
- L: Liter

The units follow a standard convention. Unless otherwise stated

- concentrations are expressed in $\frac{\mathsf{mmol}}{\mathsf{cm}^3}$
- mass fluxes in $\frac{\mathsf{mmol}}{\mathsf{cm}^3\,\mathsf{s}}$
- electric current densities in $\frac{\mu\mathsf{A}}{\mathsf{cm}^2}$
- fluxes and current densities are related by $I = z \times F \times J \times \mathcal{R}$, where z is the valence, F Faraday's constant and $\mathcal{R}$ is the volume to surface ratio.

For a clear reference, the following definitions are introduced

- [.] square bracket indicate concentrations
- $[.]_i$ the subscript i denotes internal (cytosolic) concentrations
- $[.]_o$ the subscript o denotes outer (surrounding) concentrations
- J mass fluxes
- I current densities
- I_K the subscript denotes the ion related to the current (or flux)
- $I^{Trk1,2}$ the superscript denotes the transport system related to current (or flux)
- E equilibrium potential of a transporter (Nernst potential in case of a uniport)
- E_K the subscript indicate the respecting ion for the equilibrium potential
- V_m membrane potential
- V cell volume
- A cell surface

Constants and variables

- $F = 96\,485\,000\,\mu\mathsf{C/mmol}$ Faraday's constant
- $R = 8314.5\,\mu\mathsf{J/mmol/K}$ common gas constant
- $T = 293\,\mathsf{K}$ temperature
- $R \times T = 2.4361\,10^6\,\frac{\mu\mathsf{J}}{\mathsf{mmol}}$
- z valence of a respecting ion, unless otherwise stated $z = 1$
- $F/(R \times T) = 39.6\,\frac{1}{\mathsf{V}}$
- $(R \times T)/F = 0.025\,\mathsf{V}$

- $c_m = 1 \frac{\mu F}{cm^2}$, specific membrane capacitance
- $\mathcal{R} = \frac{V}{A} = \frac{\frac{4}{3}\pi\, r^3}{4\pi\, r^2} = \frac{r}{3} \approx 7.5 \times 10^{-5}\,\mathsf{cm}$, for a spherical cell with $r = 2.28 \times 10^{-4}\mathsf{cm}$ and a volume of 50 fL
- $1/\mathcal{R} = \frac{A}{V} = \frac{3}{r} \approx 13\,000\ \ \mathsf{1/cm}$
- $\mathcal{S} = \frac{A(0)}{A}$, initial surface to actual surface ratio

Sign convention

	Current I	Flux J
Cations	< 0, influx	< 0, influx
Cations	> 0, efflux	> 0, efflux
Anions	< 0, efflux	< 0, influx
Anions	> 0, influx	> 0, efflux

Table S 1: The table gives an overview of the sign convention for the movement of anions and cations.

1.2 Kinetic model of bicarbonate reaction scheme

Transport, conversion and production of CO_2 was described by a kinetic model [12]. The bicarbonate reaction

$$CO_2 + H_2O \longleftrightarrow H_2CO_3 \longleftrightarrow HCO_3^- + H^+ \tag{1}$$

is catalyzed by carbonic anhydrase. Carbon dioxide is expected to turn quickly into carbonic acid ($pK_A = 6.35$), which dissociates into bicarbonate and protons. Since CO_2 is either present in carbonic acid or hydrogen carbonate the concentration of CO_2 can be written as

$$[CO_2] = [H_2CO_3] + [HCO_3^-]\,. \tag{2}$$

The concentrations of $[H_2CO_3]$ and $[HCO_3^-]$ can be expressed in terms of $[CO_2]$ by defining the fraction of total undissociated CO_2 as

$$\alpha = \frac{[H_2CO_3]}{[H_2CO_3] + [HCO_3^-]} = \frac{[H^+]_i}{[H^+]_i + 10^{-pK_A}}\,. \tag{3}$$

The dynamics of CO_2 is governed by

$$\frac{d}{dt}[CO_2]_i = -J_{CO_2} = -\left(J_{H_2CO_3} + J_{HCO_3^-}\right)\,, \tag{4}$$

where we have defined the reaction rates

$$J_{H_2CO_3} = \frac{P_{H_2CO_3}}{-\mathcal{R}}\left([H_2CO_3]_o - [H_2CO_3]_i\right) + J^{prod.}_{H_2CO_3} \tag{5}$$

$$J_{HCO_3^-} = \frac{P_{HCO_3^-}}{-\mathcal{R}}\frac{V_m\,F}{R\,T} \times \frac{[HCO_3^-]_o - [HCO_3^-]_i\, e^{\frac{-V_m\,F}{R\,T}}}{1 - e^{\frac{-V_m\,F}{R\,T}}} + J^{prod.}_{HCO_3^-}\,. \tag{6}$$

The production terms

$$J^{prod.}_{H_2CO_3} = \alpha \times J^{prod.}_{CO_2} \tag{7}$$

$$J^{prod.}_{HCO_3^-} = (1-\alpha) \times J^{prod.}_{CO_2}\,, \tag{8}$$

are determined by $J^{prod.}_{CO_2}$, which is an input to the model and describes the metabolic production of CO_2. This time dependent input function was inferred in the reverse tracking approach, see Section 1.4.

Collecting these terms, the dynamics of the internal pH reads

$$\frac{d}{dt}pH_i = \frac{1}{\beta}\left(J_H^{transp.} + J_H^{CO_2 react.}\right) ,$$

with $J_H^{transp.} = J_H^{Pma1} + J_H^{Nha1} + J_H^{leak}$ and $J_H^{CO_2 react.} = (1-\alpha)J_{H_2CO_3} - \alpha J_{HCO_3^-}$.

1.3 Complete model equations

The full model reads

$$\frac{d}{dt}[K^+]_i = -\left(J_K^{Trk1,2} + J_K^{Nha1} + J_K^{Tok1} + J_K^{leak}\right) \tag{9}$$

$$\frac{d}{dt}[CO_2]_i = -\left(J_{H_2CO_3} + J_{HCO_3^-}\right) \tag{10}$$

$$\frac{d}{dt}pH_i = \frac{1}{\beta}\left(J_H^{Pma1} + J_H^{Nha1} + J_H^{leak} + \right. \tag{11}$$

$$\left. + (1-\alpha)J_{H_2CO_3} - \alpha J_{HCO_3^-}\right) \tag{12}$$

$$\frac{d}{dt}V = L_p \times A \times (\Pi_i - \Pi_o - \Pi_t) . \tag{13}$$

The membrane potential is expressed by the charge balance equation [13]

$$V_m = \frac{-F \times V}{c_m \times A}\left(\beta pH_i - [K^+]_i + [HCO_3^-]_i - [Z]\right) \tag{14}$$

$$= \mathcal{F}\left(\beta pH_i - [K^+]_i + \frac{10^{-pH_i}}{10^{-pH_i} + 10^{-pK_A}}[CO_2]_i - [Z]\right) , \tag{15}$$

with Faradays constant F, specific membrane capacitance c_m, cell surface A and $[Z]$ sums up all fixed charges and is defined by the choice of $[K^+]_i(0)$, $pH_i(0)$, $V(0)$, $[CO_2]_i(0)$ and $V_m(0)$.

$$I_K^{Trk1,2} = \frac{g_{Trk1,2} \times \mathcal{S}}{1 + e^{d_{Trk1,2} \times \frac{zF}{RT}(V_m - V^{1/2}_{Trk1,2})}} \times (V_m - E_{Trk1,2}) \tag{16}$$

$$I_K^{Nha1} = -\frac{g_{Nha1} \times \mathcal{S}}{1 + e^{d_{Nha1} \times \frac{zF}{RT}(V_m - V^{1/2}_{Nha1})}} \times (V_m - E_{Nha1}) \tag{17}$$

$$I_K^{Tok1} = \frac{g_{Tok1} \times \mathcal{S}}{1 + e^{d_{Tok1} \times \frac{zF}{RT}(V_m - V^{1/2}_{Tok1})}} \times (V_m - E_{Tok1}) \tag{18}$$

$$I_K^{K,leak} = g_{K,leak} \times \mathcal{S} \times (V_m - E_{K,leak}) \tag{19}$$

$$I_H^{Pma1} = I_{Pma1}^{max} \times \mathcal{S} \times \tanh\left(\frac{zF}{2RT}\left(V_m - \left(E_H + \frac{\Delta G_{ATP}}{F}\right)\right)\right) \tag{20}$$

$$I_H^{Nha1} = -2 \times I_K^{Nha1} \tag{21}$$

$$I_H^{H,leak} = g_{H,leak} \times \mathcal{S} \times (V_m - E_{H,leak}) \tag{22}$$

$$\tag{23}$$

$$
\begin{aligned}
J_{H_2CO_3} &= \frac{P_{H_2CO_3}}{-\mathcal{R}} \left([H_2CO_3]_o - [H_2CO_3]_i\right) + J^{prod.}_{H_2CO_3} && (24)\\
J_{HCO_3^-} &= \frac{P_{HCO_3^-}}{-\mathcal{R}} \frac{V_m\,F}{R\,T} \times \frac{[HCO_3^-]_o - [HCO_3^-]_i\, e^{\frac{-V_m\,F}{R\,T}}}{1 - e^{\frac{-V_m\,F}{R\,T}}} + J^{prod.}_{HCO_3^-} && (25)\\
J_H^{CO_2 react.} &= (1-\alpha) J_{H_2CO_3} - \alpha J_{HCO_3^-} && (26)\\
J^{prod.}_{H_2CO_3} &= \alpha \times J^{prod.}_{CO_2} && (27)\\
J^{prod.}_{HCO_3^-} &= (1-\alpha) \times J^{prod.}_{CO_2} && (28)\\
\alpha &= \frac{[H^+]_i}{[H^+]_i + 10^{-pK_A}} && (29)\\
& && (30)
\end{aligned}
$$

$$
\begin{aligned}
\Pi_o &= \big([K^+]_o + [H^+]_o + [Cl^-]_o + [CO_2]_o + [X]_o\big) \times R\,T && (31)\\
\Pi_i &= \frac{V}{V - f_V \times V(0)} \times \big([K^+]_i + [H^+]_i + [CO_2]_i + [X]_i\big) \times R\,T && (32)\\
\Pi_t &= \Pi_t(0) \times \frac{V - k_V \times V(0)}{V(0) - k_V \times V(0)} && (33)\\
\Pi_t(0) &= \Pi_i(0) - \Pi_o(0)\,. && (34)
\end{aligned}
$$

The production rate $J^{prod.}_{CO_2}$ is an input to the model, see Sections 1.2 and 1.4. Note that the production changes with the volume even if the amount of produced CO_2 does not change. This is taken into account by the relationship

$$
J^{prod.}_{CO_2} = \tilde{J}^{prod.}_{CO_2} \times \frac{V(0)}{V(t)}\,, \tag{35}
$$

where $V(0)$ denotes the initial volume. Similarly, the factor $\mathcal{S} = \frac{A(0)}{A(t)}$ was introduced to correctly cover the surface dependence of the conductance parameters.

The equilibrium potentials are given as

$$
\begin{aligned}
E_{Trk1,2} = E_{K,leak} = E_{Tok1} &= \frac{RT}{zF} \ln \frac{[K^+]_o}{[K^+]_i} && (36)\\
E_{Nha1} &= \frac{RT}{zF} \ln \left(\frac{[K^+]_i}{[K^+]_o} \times \frac{[H^+]_o^2}{[H^+]_i^2} \right) && (37)\\
E_{H,leak} = E_H &= \frac{RT}{zF} \ln \frac{[H^+]_o}{[H^+]_i}\,. && (38)
\end{aligned}
$$

The starvation process (reduction of external KCl) is modeled as

$$
[K^+]_o(t) = [Cl^-]_o(t) = \begin{cases} 0.05 \frac{\mathsf{mmol}}{\mathsf{cm}^3}, & \text{for } t < 0\,sec,\\ 15 \times 10^{-6} + \big(0.05 - 15 \times 10^{-6}\big) \times \exp\left(-\left(\frac{t-\mu}{\sigma}\right)^2\right) \frac{\mathsf{mmol}}{\mathsf{cm}^3}, & \text{otherwise} \end{cases} \tag{39}
$$

where $\mu = 0$ and $\sigma = 0.04 \times 60^2\,\mathsf{s}$.

1.4 Reverse Tracking Approach

The model equations in Section 1.3 are thermodynamically consistent but do not account for any regulatory events (e.g. gene regulation or signal transduction) which might modulate the activity of the transport channels and other components of the system. We observed that extensive parameter scans did not yield a satisfactory fit to the observed time course for intracellular potassium (see Figure 1A in the main text and Section 2). On the one hand this indicates that the model is not "overfitted" but on the other hand it shows that important aspects of the homeostatic response are not covered by the model. Potassium homeostasis involves a variety of cellular and metabolic processes. Exhaustive modeling of all these processes is currently impossible due to lack of detailed knowledge and lack of experimental data. Instead we decided to treat parameters of the models as unknown input functions which are modulated by these unmodeled processes. We used the parameters determining the activity of the transport systems and the carbon dioxide production rate as potentially altered by unmodeled external processes. Instead of estimating a number for this parameter we tried to infer a time course for this input function ("time dependent parameter") by minimizing an objective function which measures the difference between the data and the simulation. In particular, we solved the minimization problem

$$\min_{p(t)} ||[K^+]_i^{sim}(p(t),\theta) - [K^+]_i^{exp}|| \,. \tag{40}$$

Here, $[K^+]_i^{exp}$ and $[K^+]_i^{sim}(p(t),\theta)$ denote the experimental and the simulated potassium concentration respectively. The latter depends on the time dependent parameter $p(t)$ which is tested and further constant parameters θ. Each transport system or component in the model for which we were able to find a $p(t)$ with a satisfactory minimum were considered as potential actuators of potassium homeostasis.

The numerical problem to estimate a time course, which tracks the simulated potassium along a given path can be solved by the implementation of an I-controller

$$\frac{d}{dt}p(t) = \eta \times \left([K^+]_i^{sim}(p(t),\theta) - [K^+]_i^{data}\right) \,, \tag{41}$$

with an appropriate amplification factor η. It was tentatively chosen in each individual case to reduce oscillations possibly produced by the I-controller.

The specific reverse tracking approaches for carbon dioxide production and the regulation of transport systems are given below.

Carbon dioxide production

For the carbon dioxide production rate we used

$$\frac{d}{dt}\tilde{J}_{CO_2}^{prod.}(t) = \eta_{CO_2} \times \left([K^+]_i^{sim}(\tilde{J}_{CO_2}^{prod.}(t),\theta) - [K^+]_i^{data}\right) \tag{42}$$

$$J_{CO_2}^{prod.}(t) = \tilde{J}_{CO_2}^{prod.}(t) \times \frac{V(0)}{V(t)} \,, \tag{43}$$

where the volume dependent production rate was scaled by the initial volume to the actual volume ratio.

Transport systems

In case of the transport systems we used $p(t)$ as a kind of regulation function for a constant conductivity parameter $\tilde{g}$:

$$\frac{d}{dt}p(t) = \eta \times \left([K^+]_i^{sim}(p(t),\theta) - [K^+]_i^{data}\right) \tag{44}$$

$$g(t) = p(t) \times \tilde{g} \,, \tag{45}$$

where $g(t)$ gives the time dependent conductance parameter.

2 Supplementary Results

2.1 Trk1,2 is not a potential actuator

A priory we expected the main potassium uptake systems Trk1,2 to be potential actuators of potassium homeostasis.

For the reverse tracking approach, three different cases were analyzed:

- Trk1,2 as a K^+ uniport (model standard)
- Trk1,2 as a $2K^+$ symport
- Trk1,2 as a $1H^+/1K^+$ symport

The respective parameter values for the tested approaches are given in Supplementary Table 2.7.

The results for each case were very similar (see Supplementary Figure 1a). The loss of external potassium (traces left were 15 μM) at time point zero induces a tremendous increase (magnitude 10^4) of the conductance. However, the increased uptake rate can not compensate for the rapid loss of internal potassium (Supplementary Figure 1 b). Surprisingly, the system is not very sensitive to changes in $g_{Trk1,2}$. The incapability of tracking the potassium data led to the exclusion of Trk1,2 as a potential actuator of potassium homeostasis.

As an explanation of this finding, we suggest, that the micro-molar concentration of potassium in the medium is not enough to ensure the potassium supply. Literally spoken: opening the doors is of no use, if nothing is outside.

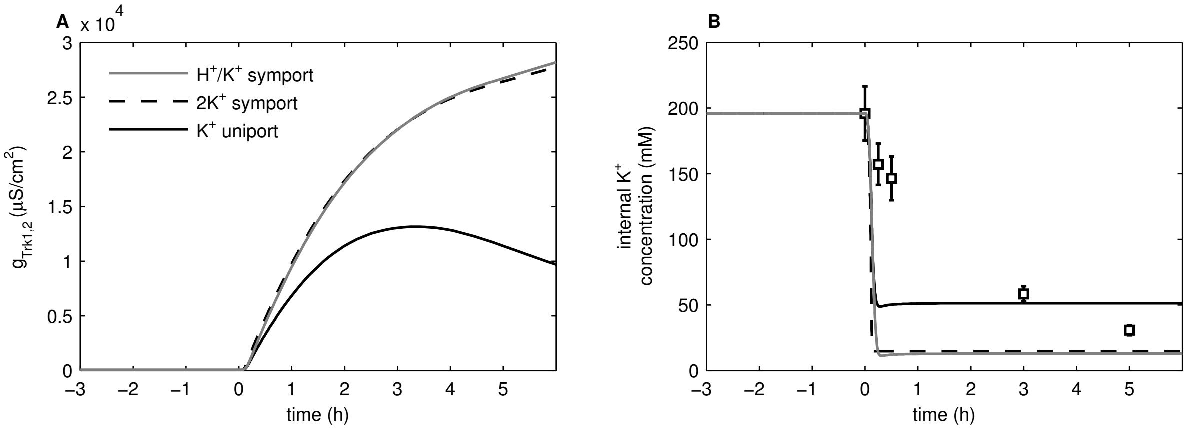

Figure S 1: **Estimated control signal for Trk1,2.** The left panel shows the estimated conductivity of Trk1,2 and the right panel shows the corresponding data fit. No tracking signal was found for any of these three cases tested.

2.2 Nha1 is not a potential actuator

For Nha1 four different possibilities were tested with the reverse tracking approach:

- Nha1 as a $2H^{+}$:$1K^{+}$ anti-porter described by Ohm's law with a voltage gating (model standard)
- Nha1 as a $2H^{+}$:$1K^{+}$ anti-porter described by Ohm's law without gating
- Nha1 as a $2H^{+}$:$1K^{+}$ anti-porter described due to Ohm's law with a potassium gating applied
- Nha1 as a $1H^{+}$:$1K^{+}$ anti-porter.

The potassium gating was modeled as a potassium dependent activation term

$$f([K^{+}]_i) = \frac{1}{1 + \frac{q_{Nha1}}{[K^{+}]_i}} \,, \tag{46}$$

which is multiplied by the constant maximum conductivity g_{Nha1}. The parameter q_{Nha1} determines the half maximal activation. The values used in Supplementary Figure 2 are given in Supplementary Table 5.

For each of the four tested model variants we found a tracking signal. However, the estimated control signal is not very plausible. At the moment of the external potassium drop, Nha1 is rapidly deactivated to prevent the potassium loss. This is followed by a phase where Nha1 is reactivated resulting in increased loss of potassium (see Supplementary Figure 2).

The time course for the wild-type, the *trk1,2* and the *nha1* mutant have the same characteristics (Figure 1A in the main text): There is an initial phase of rapid potassium loss (approx. 1h) which is followed by a second phase of slow potassium loss before a new stationary state is attained. These characteristic phases remain visible upon deletion of Nha1. From this we inferred that a supposed deactivation of Nha1 is unlikely to be the actuating process for potassium homeostasis.

In addition, the predicted increase in Nha1-activity under starvation is in contradiction to experimental findings. It was reported that the potassium content in starved cells for the wild-type, *nha1* mutants and a strain over-expressing Nha1 attains the same value after six hours of starvation [14]. This implies a deactivation of Nha1 under potassium starvation.

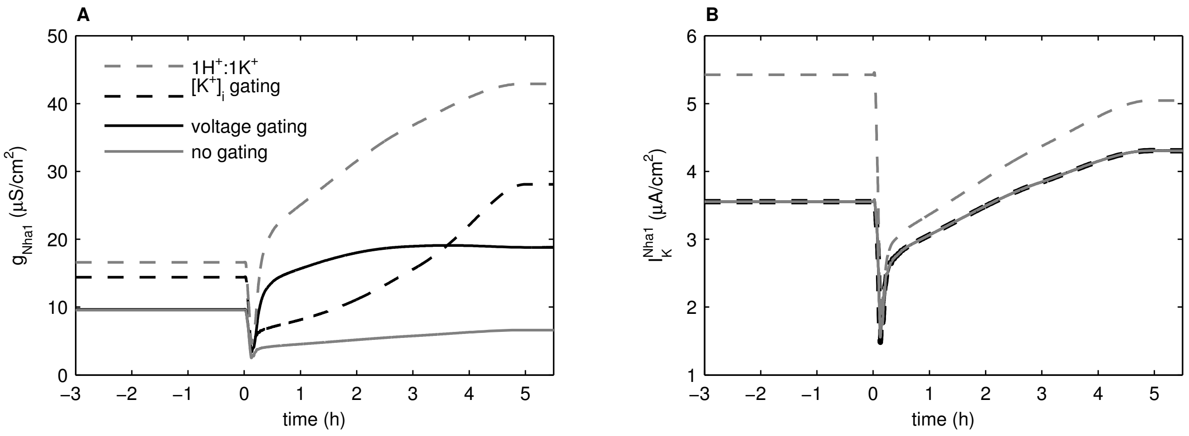

Figure S 2: **Estimated control signal for Nha1.** The left panel shows the estimated conductivity and the right panel shows the simulated Nha1 current. In each case, Nha1 is quickly deactivated and later reactivated again. This is contradictory to experimental findings [14].

2.3 Simulated membrane potential

In the main text we presented estimated CO_2 production time courses for the wild-type and for *trk1,2* mutants (see Figure 2C). The time course of the membrane potential for this estimate is shown in Supplementary Figure 3. In the simulation the removal of external potassium causes a hyperpolarization of the membrane potential. After some hours the membrane potential attains a new steady state value which is higher in magnitude compared to the pre-starvation level. In addition, the membrane potential of the *trk1,2* mutant is higher (in magnitude) than the membrane potential of the wild type. For comparison with original data see [15].

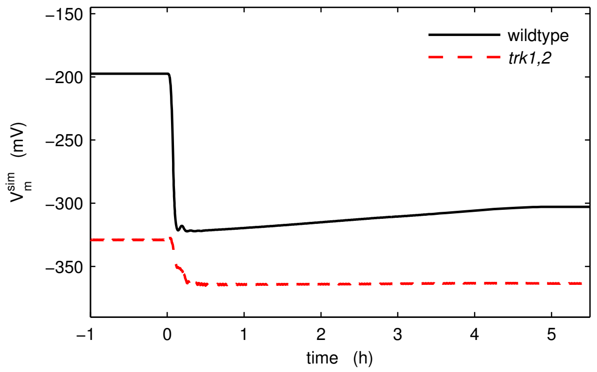

Figure S 3: **Simulated membrane potential in the reverse tracking approach.** The simulated membrane potential shows a rapid hyperpolarization at the moment of the external potassium drop at time point zero, both in the wild-type and in the *trk1,2* mutants. The latter shows a more negative membrane potential compared to the wild-type at any time, what agrees qualitatively with experimental observations [15].

2.4 Effect of Nha1 voltage gating

Electro-physiological data for Nha1 was not available. In the course of model development we started with a constant conductivity for this transporter. It turned turned out that under this assumption all potassium is lost completely, see Supplementary Figure 4. Thus we concluded, that Nha1 must be deactivated under potassium starvation in order to prevent an excessive potassium efflux. This assumption is supported by experimental observations [14] that Nha1 has an influence on the potassium content in later stages of starvation. The deactivation of Nha1 was modeled as voltage dependent gating. We observed positive stationary concentrations after starvation. Nevertheless, deactivation of Nha1 is not sufficient to reproduce the slow dynamics of potassium loss after starvation.

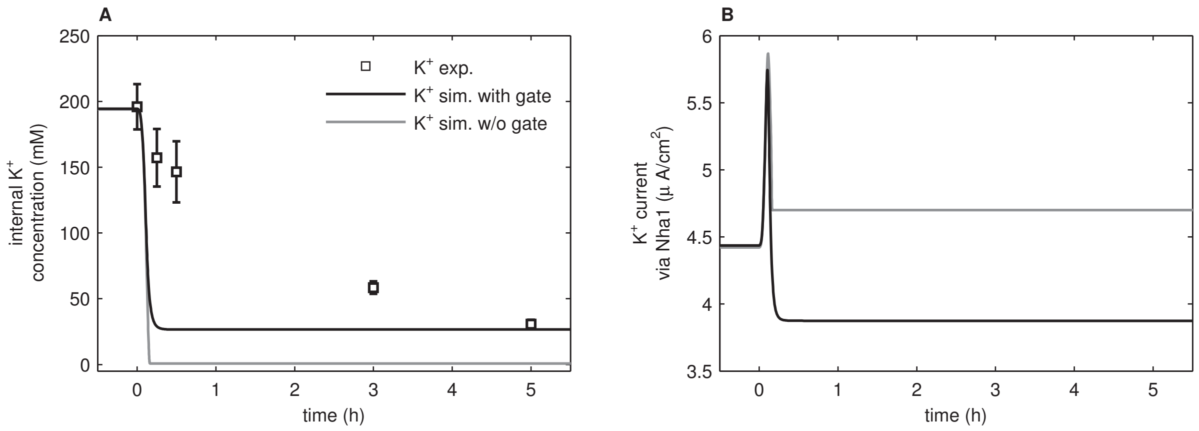

Figure S 4: **Nha1 voltage gating.** Simulated potassium starvation with and without a voltage gating of Nha1. Left panel: the error-bars gives the raw data from the literature [15], the black line is the simulated potassium concentration with Nha1 voltage gating and the gray line is the simulation without voltage gating. The external potassium drop occurs at time point zero. Without a voltage gating, potassium is completely lost after the external potassium drop. Right panel: simulated Nha1 potassium current. In case of the Nha1 voltage gating the current finally reaches a lower stationary value compared to a constant conductivity model. Voltage gating can limit the potassium efflux, but it is not sufficient to reproduce the slow dynamics of potassium loss after starvation.

2.5 Simulated volume experiment

The potassium starvation experiment of [15] also comprises the measurement of the cell volume. Supplemental Figure 5 gives the respective measurements and model simulations for the wildtype and the *trk1,2* mutant. In both cases, the volume decreases after the shift to the potassium free medium. However, the volume decrease is higher in the wildtype. As it was shown in Figure 1A the *trk1,2* mutant maintains a higher potassium concentration compared to the wildtype. Thus, the volume measurement highlights the role of potassium as a major osmotic substance.

However, the high variability of the cell volume in the measurements indicate the involvement of other processes like cell growth and division or volume regulation. These processes are not characterized for the given data and are not included in the volume description. Supplementary Figure 5 shows the simulated volume for the reverse tracking approach from Figure 2B. The simulated volume is only in qualitative agreement with the measurements. This indicates, that the volume model needs further improvements. However, we found the volume of minor relevance since even the assumption of a constant volume does not lead to a visible change in the overall result.

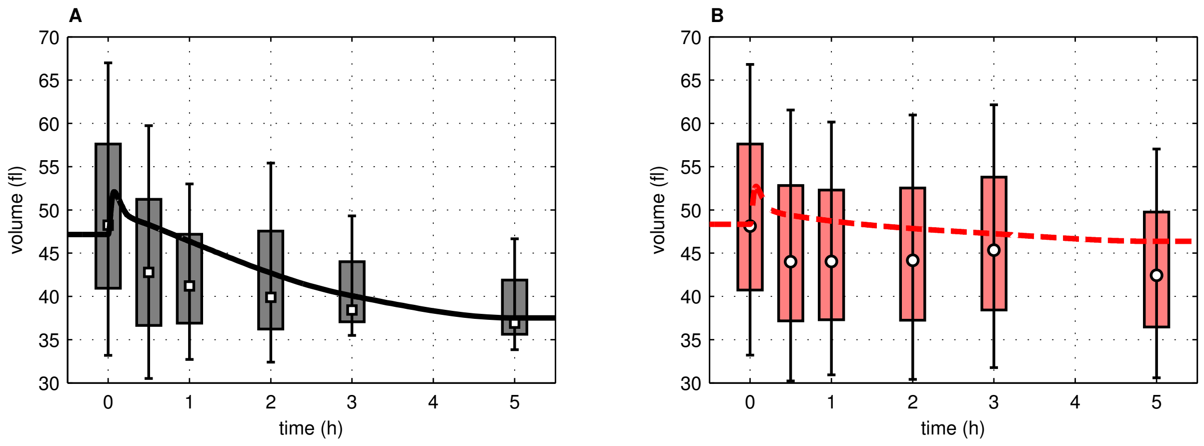

Figure S 5: **Simulated volume experiment.** The volume measurements of [15] show a high natural variability. The simulated volume is in qualitative agreement with the data.

2.6 Data fit of Trk1,2

In the simplest case a transport systems behaves like an ohmic resistance and electrophysiological measurements show a liner dependence between membrane voltage and current density. A related approach is the voltage gating, where the conductance parameter itself is dependent on the membrane voltage. The so called Boltzmann function or Boltzmann expression can be derived from the voltage dependent transition between an active and inactive state of the transporter [1–5]

$$g(V_m) = \frac{g}{1 + e^{d \cdot \frac{zF}{RT}\left(V_m - V^{1/2}\right)}} \,, \tag{47}$$

with constant conductivity g, the parameter d controlling the steepness of the transition and $V^{1/2}$ is the membrane potential of half maximal activation. This expression can be interpreted as the multiplication of a constant conductivity with a voltage dependent probability for the transporter to be active.

The paper from [6] Figure 9c, gives the current-voltage relationship for the main potassium uptake system Trk1,2. The measurements are performed for both Trk proteins together and do not allow for a distinction between Trk1 and Trk2; raw data see Figure 6. The electro-physiological measurements were obtained under four different conditions. The external KCl concentration was either 150 mM, 10 mM, 1 mM or 50 μM and the internal potassium concentrations was 30 mM in all four cases.

Using Matlab and its `lsqnonlin` procedure, we fitted the expression

$$I = \frac{g}{1 + e^{d \cdot \frac{zF}{RT}\left(V_m - V^{1/2}\right)}} \left(V_m - \frac{RT}{zF} \ln \frac{[K^+]_o}{[K^+]_i} \right) \,, \tag{48}$$

to the data of [6] Figure 9c. This yielded one set of parameters $\{g,\, d,\, V^{1/2}\}$ which accurately describes each of the four data sets; see Supplementary Figure 6.

$$g_{Trk1,2} \;=\; 22.28\,\frac{\mu\mathsf{S}}{\mathsf{cm}^2} \tag{49}$$

$$d_{Trk1,2} \;=\; 0.99 \tag{50}$$

$$V^{1/2}_{Trk1,2} \;=\; -0.168\,\mathsf{V}\,. \tag{51}$$

The nonlinear fit was robust to different initial values or parameter boundaries of the fitting routine.

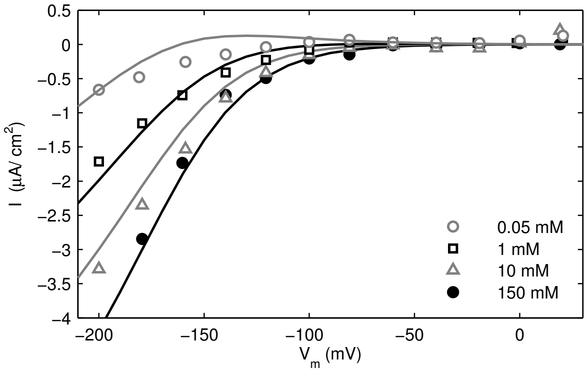

Figure S 6: **Gated channel approach for Trk1,2.**The figure visualizes the data of [6], Fig. 9c and the respective data fit according to the gated channel approach; Equation (48). Only three different parameters were needed to describe the four datasets at once.

2.7 Nha1 data

Figure 1A in the main text shows two repetitions of a potassium starvation experiment with *nha1* mutants. To distinguish between the two experiments we present this data again in Supplementary Figure 7. In tendency, the potassium reduction is fast in the first hour of starvation and slows down later on.

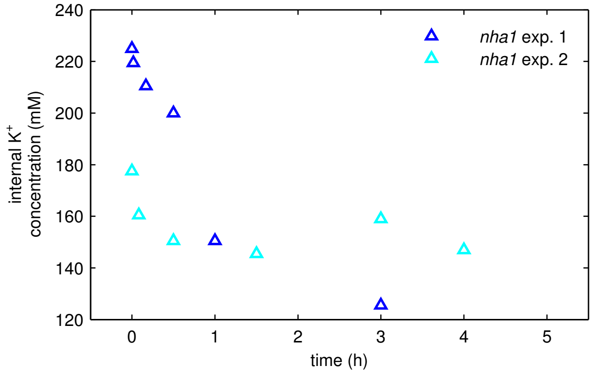

Figure S 7: **Potassium starvation experiment with *nha1* mutants.** For clarity we show the two repetitions from Figure 1A separately.

Supplementary Tables

Variable	Value	Unit	Description	Reference
c_m	1	μF/cm^2	specific membrane capacitance	[3]
β	0.2	mmol/cm^3/pH	H^+ buffer capacity	based on [13,16]
$g_{Trk1,2}$	22.28	μS/cm^2	conductance Trk1,2	data fit [6]
$d_{Trk1,2}$	1	dimensionless	voltage sensitivity	data fit [6]
$V_{1/2,Trk1,2}$	-0.168	V	half maximal voltage	data fit [6]
g_{Tok1}	9	μS/cm^2	conductance Tok1	[7]
d_{Tok1}	-1	dimensionless	voltage sensitivity	[7]
V_{Tok1}	-0.0034	V	half maximal voltage	[7]
g_{Nha1}	8-12	μS/cm^2	conductance Nha1	tentative
d_{Nha1}	1	dimensionless	voltage sensitivity	tentative
$V_{1/2,\,Nha1}$	-0.300	V	half maximal voltage	tentative
I_{Pma1}^{max}	16	μA/cm^2	maximal pump current	based on [8,10]
ΔG_{ATP}	-40×10^6	μJ/mmol	energy ATP hydrolysis	based on [10]
g_K^{leak}	5	μS/cm^2	conductance	data fit [6]
g_H^{leak}	25	μS/cm^2	conductance	[8]
$P_{H_2CO_3}$	6×10^{-3}	cm/s	permeability	[12]
$P_{HCO_3^-}$	5×10^{-7}	cm/s	permeability	[12]
pK_A	6.35	dimensionless	acidity constant CO_2	[17]
$[K^+]_o$	0.05 to 15×10^{-6}	mmol/cm^3	external potassium	[15]
$[Cl^-]_o$	0.05 to 15×10^{-6}	mmol/cm^3	external potassium	[15]
pH_o	5.8	pH	external pH	[15]
L_p	4×10^{-11}	cm^4/μJ/s	hydraulic conductivity	[11]
f_V	0.99	dimensionless	elasticity of the cell wall	[11]
k_V	0.37	dimensionless	fraction of non-osmotic volume	[11]
$[X]_o$	0.15	mmol/cm^3	osmotic active conc. extern	[15]
$[X]_i$	0.3	mmol/cm^3	osmotic active conc. intern	based on [11]

Table S 2: Suitable values and respecting sources, which can serve as an orientation in the parameter space. The simulation of a certain experiment requires an adoption of the parameter values.

Variable	wt, CO_2	wt, Pma1	*trk1,2*, CO_2,	*trk1,2*, Pma1	unit
$[K^+]_i(0)$	0.2	0.2	0.2	0.2	$\frac{mmol}{cm^3}$
$[CO_2]_i(0)$	0.01	0.01	0.01	0.01	$\frac{mmol}{cm^3}$
$pH_i(0)$	7	7	6.5	6.5	pH
$V_m(0)$	-0.2	-0.2	-0.2	-0.2	V
$V(0)$	48e-12	48e-12	48e-12	48e-12	cm^3
$J_{CO_2}^{prod}(0)$	5e-4	n.a.	5e-4	n.a.	$\frac{mmol}{cm^3 \times s}$
$\tilde{I}_{Pma1}^{max}$	n.a.	15	n.a.	15	$\frac{\mu A}{cm^2}$
$p_{Pma1}(0)$	n.a.	1	n.a.	1	/
$\tilde{g}_{Trk1,2}$	23	n.a.	23	n.a.	$\frac{\mu S}{cm^2}$
$p_{Trk1,2}(0)$	1	n.a.	1	n.a.	/
pH_o	5.8	5.8	5.8	5.8	pH
$[CO_2]_o$	1e-3	1e-3	1e-3	1e-3	$\frac{mmol}{cm^3}$
η	-1e-3	-1	-1e-2	-120	/
c_m	1	1	1	1	$\frac{\mu F}{cm^2}$
β	0.2	0.2	0.2	0.2	$\frac{mmol}{pH \times cm^3}$
$g_{Trk1,2}$	23	23	23.	23	$\frac{\mu S}{cm^2}$
$d_{Trk1,2}$	1	1	1	1	/
$V_{1/2,Trk1,2}$	-0.168	-0.168	-0.168	-0.168	V
g_{Tok1}	9	9	9	9	$\frac{\mu S}{cm^2}$
d_{Tok1}	-1	-1	-1	-1	/
V_{Tok1}	-0.0034	-0.0034	-0.0034	-0.0034	V
g_{Nha1}	10	10	10	10	$\frac{\mu S}{cm^2}$
d_{Nha1}	-1	-1	-1	-1	/
V_{Nha1}	-0.31	-0.31	-0.31	-0.31	V
I_{Pma1}^{max}	15	n.a.	15	n.a.	$\frac{\mu A}{cm^2}$
ΔG_{ATP}	-40e+6	-40e+6	-40e+6	-40e+6	$\frac{kJ}{mmol}$
g_K^{leak}	5	5	5	5	$\frac{\mu S}{cm^2}$
g_H^{leak}	25	25	25	25	$\frac{\mu S}{cm^2}$
$P_{H_2CO_3}$	6e-3	6e-3	6e-3	6e-3	$\frac{cm}{s}$
$P_{HCO_3^-}$	5e-7	5e-7	5e-7	5e-7	$\frac{cm}{s}$
$J_{CO_2}^{prod}$	n.a.	1e-4	n.a.	1e-4	$\frac{mmol}{cm^3 \times s}$
pK_A	6.35	6.35	6.35	6.35	/
L_p	4e-11	4e-11	4e-11	4e-11	$\frac{cm^4}{s \times \mu J}$
k_V	0.5	0.5	0.5	0.5	/
f_V	0.37	0.37	0.37	0.37	/
$[X]_o$	0.15	0.15	0.15	0.15	$\frac{mmol}{cm^3}$
$[X]_i$	0.2	0.2	0.2	0.2	$\frac{mmol}{cm^3}$

Table S 3: Reverse tracking approach: Parameters used to estimate the time courses of CO_2 production and Pma1 regulation for the potassium starvation in wild-type and *trk1,2*. These parameters were used to generate the Figures 2B,C of the print version and also to simulate the membrane potential in Supplementary Figure 3.

Variable	wt, Trk1,2, K^+	wt, Trk1,2, $2K^+$	wt, Trk1,2, H^+/K^+	unit
$[K^+]_i(0)$	0.2	0.2	0.2	$\frac{mmol}{cm^3}$
$[CO_2]_i(0)$	0.01	0.01	0.01	$\frac{mmol}{cm^3}$
$pH_i(0)$	7	7	7	pH
$V_m(0)$	-0.2	-0.2	-0.2	V
$V(0)$	48e-12	48e-12	48e-12	cm^3
$\tilde{g}_{Trk1,2}$	23	23	23	$\frac{\mu S}{cm^2}$
$p_{Trk1,2}(0)$	1	1	1	/
pH_o	5.8	5.8	5.8	pH
$[CO_2]_o$	1e-3	1e-3	1e-3	$\frac{mmol}{cm^3}$
η	-1	-1	-1	/
c_m	1	1	1	$\frac{\mu F}{cm^2}$
β	0.2	0.2	0.2	$\frac{mmol}{pH \times cm^3}$
$g_{Trk1,2}$	n.a.	n.a.	n.a.	$\frac{\mu S}{cm^2}$
$d_{Trk1,2}$	1	1	1	/
$V_{1/2,Trk1,2}$	-0.168	-0.168	-0.168	V
g_{Tok1}	9	9	9	$\frac{\mu S}{cm^2}$
d_{Tok1}	-1	-1	-1	/
V_{Tok1}	-0.0034	-0.0034	-0.0034	V
g_{Nha1}	10	10	10	$\frac{\mu S}{cm^2}$
d_{Nha1}	-1	-1	-1	/
V_{Nha1}	-0.31	-0.31	-0.31	V
I^{max}_{Pma1}	15	15	15	$\frac{\mu A}{cm^2}$
ΔG_{ATP}	-40e+6	-40e+6	-40e+6	$\frac{kJ}{mmol}$
g^{leak}_K	5	5	5	$\frac{\mu S}{cm^2}$
g^{leak}_H	25	25	25	$\frac{\mu S}{cm^2}$
$P_{H_2CO_3}$	6e-3	6e-3	6e-3	$\frac{cm}{s}$
$P_{HCO_3^-}$	5e-7	5e-7	5e-7	$\frac{cm}{s}$
$J^{prod}_{CO_2}$	1e-4	1e-4	1e-4	$\frac{mmol}{cm^3 \times s}$
pK_A	6.35	6.35	6.35	/
k_B	$10^{-6.5}$	$10^{-6.5}$	$10^{-6.5}$	/
L_p	4e-11	4e-11	4e-11	$\frac{cm^4}{s \times \mu J}$
k_V	0.5	0.5	0.5	/
f_V	0.37	0.37	0.37	/
$[X]_o$	0.15	0.15	0.15	$\frac{mmol}{cm^3}$
$[X]_i$	0.2	0.2	0.2	$\frac{mmol}{cm^3}$

Table S 4: Reverse tracking approach: Parameters used to estimate the time courses of CO_2 production for the potassium starvation in the wild-type. These parameters were used to generate Supplementary Figure 1.

Variable	Nha1	Nha1 K^+ gating	Nha1 no gating	Nha1 $1H^+$:$1K^+$	unit
$[K^+]_i(0)$	0.2	0.2	0.2	0.2	$\frac{mmol}{cm^3}$
$[CO_2]_i(0)$	0.01	0.01	0.01	0.01	$\frac{mmol}{cm^3}$
$pH_i(0)$	7	7	7	7	pH
$V_m(0)$	-0.2	-0.2	-0.2	-0.2	V
$V(0)$	48e-12	48e-12	48e-12	48e-12	cm^3
$\tilde{g}_{Nha1}$	10	10	10	10	$\frac{\mu S}{cm^2}$
$p_{Nha1}(0)$	1	1	1	1	/
pH_o	5.8	5.8	5.8	5.8	pH
$[CO_2]_o$	1e-3	1e-3	1e-3	1e-3	$\frac{mmol}{cm^3}$
η	2	2	2	2	/
c_m	1	1	1	1	$\frac{\mu F}{cm^2}$
β	0.2	0.2	0.2	0.2	$\frac{mmol}{pH \times cm^3}$
$g_{Trk1,2}$	23	23	23	23	$\frac{\mu S}{cm^2}$
$d_{Trk1,2}$	1	1	1	1	/
$V_{1/2,Trk1,2}$	-0.168	-0.168	-0.168	-0.168	V
g_{Tok1}	9	9	9	9	$\frac{\mu S}{cm^2}$
d_{Tok1}	-1	-1	-1	-1	/
V_{Tok1}	-0.0034	-0.0034	-0.0034	-0.0034	V
g_{Nha1}	n.a.	n.a.	n.a.	n.a.	$\frac{\mu S}{cm^2}$
d_{Nha1}	-1	n.a.	n.a.	-1	/
V_{Nha1}	-0.31	n.a.	n.a.	-0.31	V
q_{Nha1}	n.a.	0.1	n.a.	n.a.	$\frac{mmol}{cm^3}$
I^{max}_{Pma1}	15	15	15	15	$\frac{\mu A}{cm^2}$
ΔG_{ATP}	-40e+6	-40e+6	-40e+6	-40e+6	$\frac{kJ}{mmol}$
g^{leak}_K	5	5	5	5	$\frac{\mu S}{cm^2}$
g^{leak}_H	25	25	25	25	$\frac{\mu S}{cm^2}$
$P_{H_2CO_3}$	6e-3	6e-3	6e-3	6e-3	$\frac{cm}{s}$
$P_{HCO_3^-}$	5e-7	5e-7	5e-7	5e-7	$\frac{cm}{s}$
$J^{prod}_{CO_2}$	1e-4	1e-4	1e-4	1e-4	$\frac{mmol}{cm^3 \times s}$
pK_A	6.35	6.35	6.35	6.35	/
L_p	4e-11	4e-11	4e-11	4e-11	$\frac{cm^4}{s \times \mu J}$
k_V	0.5	0.5	0.5	0.5	/
f_V	0.37	0.37	0.37	0.37	/
$[X]_o$	0.15	0.15	0.15	0.15	$\frac{mmol}{cm^3}$
$[X]_i$	0.2	0.2	0.2	0.2	$\frac{mmol}{cm^3}$

Table S 5: Reverse tracking approach: Parameters used to estimate the time courses of CO_2 production for the potassium starvation in the wild-type. These parameters were used to generate Supplementary Figure 2.

Supplementary References

1. Weiss TF (1996) Cellular Biophysics - Transport. Bradford.
2. Weiss TF (1996) Cellular Biophysics - Electrical properties. Bradford.
3. Hille B (2001) Ion channels of excitable membranes. Sinauer.
4. Fall C (2002) Computational Cell Biology. Berlin: Springer.
5. Keener J, Sneyd J (2004) Mathematical Physiology. Springer.
6. Kuroda T, Bihler H, Bashi E, Slayman C, Rivetta A (2004) Chloride channel function in the yeast Trk-potassium transporters. Journal of Membrane Biology 198: 177-192.
7. Johansson I, Blatt M (2006) Interactive domains between pore loops of the yeast K^+ channel Tok1 associate with extracellular K^+ sensitivity. Bioche J 393: 645-655.
8. Sanders D, Hansen U, Slayman C (1981) Role of the plasma membrane proton pump in pH regulation in non-animal cells. Proc Natl Acad Sci USA 78: 5903-5907.
9. Endresen LP, Hall K, Hoye JS, Myrheim J (2000) A theory for the membrane potential of living cells. Eur Biophys J 29: 90-103.
10. Gradmann D, Hansen U, Long WS, Slayman C, Warncke J (1978) Current voltage relationships for the plasma membrane and its principal electrogenic pump in *Neurospora crassa*: I. Steady-state conditions. Journal of Membrane Biology 39: 333-367.
11. Gennemark P, Nordlander B, Hohmann S, Wedlin D (2006) A simple mathematical model of adaption to high osmolarity in yeast. In silico Biology 6: 34.
12. Boron W, de Weer P (1976) Intracellular pH transients in squid giant axons caused by CO_2, NH_3 and metabolic inhibitors. The Journal of General Physiology 67: 91-112.
13. Grabe M, Oster G (2001) Regulation of organell acidity. J Gen Physiol 117: 329-343.
14. Bañuelos M, Ruiz M, Jiménez A, Souciet JL, Potier S, et al. (2002) Role of the Nha1 antiporter in regulating K^+ influx in Saccharomyces cerevisiae. Yeast 19: 9-15.
15. Navarrete C, Petrezsélyová S, Barreto L, Martínez J, Zahrádka J, et al. (2010) Lack of main K^+ uptake systems in S. cerevisiae cells affects yeast cell physiological parameters both in potassium sufficient and limiting conditions. FEMS Yeast Res 10: 508-517.
16. Blatt M, Slayman C (1987) Role of active potassium transport in the regulation of cytoplasmic pH by nonanimal cells. Proc Natl Acad Sci USA 84: 2737-2741.
17. Lopéz R, Enríquez E, Peña A (1999) Effects of weak acids on cation accumulation, ΔpH and $\Delta\Psi$ in yeast. YEAST 15: 553562.
